# Single cell and spatial transcriptomics in human tendon disease indicate dysregulated immune homeostasis

**DOI:** 10.1136/annrheumdis-2021-220256

**Published:** 2021-05-17

**Authors:** Moeed Akbar, Lucy MacDonald, Lindsay A N Crowe, Konstantin Carlberg, Mariola Kurowska-Stolarska, Patrik L Ståhl, Sarah J B Snelling, Iain B McInnes, Neal L Millar

**Affiliations:** 1 Institute of Infection, Immunity and Inflammation, College of Medical Veterinary and Life Sciences, University of Glasgow, Glasgow, UK; 2 Research into Inflammatory Arthritis Centre Versus Arthritis (RACE), University of Glasgow, Glasgow, UK; 3 Science for Life Laboratory, Dept. of Gene Technology, KTH Royal Institute of Technology, Solna, Sweden; 4 Nuffield Department of Orthopaedics, Rheumatology and Musculoskeletal Sciences, University of Oxford, Oxford, UK

**Keywords:** tendinopathy, inflammation, arthritis

Tendinopathy; encompassing multifactorial tendon disorders characterised by pain and functional limitation remains a significant burden in musculoskeletal medicine.^1^ Recent findings highlight a key role for immune mediated mechanisms in tendon disease supporting the concept that pivotal immunological and biomechanical factors conventionally associated with inflammatory rheumatic and musculoskeletal diseases (RMDs) are manifest in tendon.^2^ Single cell technologies^3^ (scRNAseq) are increasingly applied in rheumatology to identify key cellular phenotypes that drive disease pathogenesis. Despite efforts with small cell numbers and heterogenous tendon biopsies^4^ there remains no detailed spatial tendon cell atlas to inform translational targeting. Herein, for the first time utilising scRNAseq and spatial transcriptomics (S_T_), we carry out cell–cell interaction analysis to build an atlas of the dynamic cellular environment that drives the development of chronic human tendon disease.

**Figure 1:**
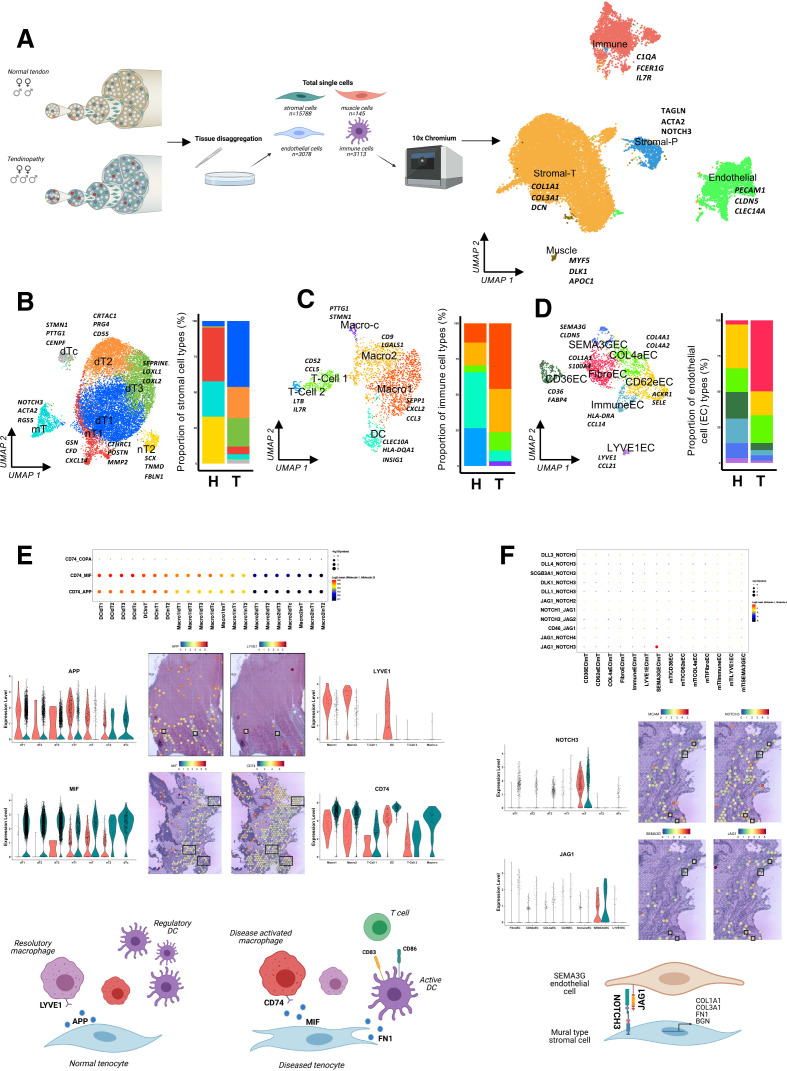
Cell composition and interactions of healthy and diseased human tendon. (A) Normal (n=4, human hamstring tendon) and diseased (tendinopathy, n=5, human supraspinatus tendon) human tendon were processed for single cell analysis using Chromium 10x 3′DEG chemistry. Infographic shows number of donors and cells sequenced. Uniform manifold approximation and projection (UMAP) embedding of 22 124 single cells delineating endothelial, immune, stromal tenocyte and stromal mural cells with marker genes. (B) Stromal cells of the tendon. UMAP embedding with gene markers and distribution of seven delineated stromal cell populations from human tendons; mural tenocyte (mT), normal tenocyte1 (nT1), normal tenocyte2 (nT2), diseased tenocyte1 (dT1), diseased tenocyte2 (dT2), diseased tenocyte3 (dT3) and diseased cycling tenocytes (dTc). (C) Immune cells of the tendon. UMAP embedding with gene markers and distribution of 6 delineated immune cell populations from human tendons; dendritic cells (DC), macrophage1 (Macro1), macrophage1 (Macro2), cycling macrophage (Macro-C), T-Cells1 (T-Cell1) and T-Cells2 (T-Cell2). (D) Endothelial cells (EC) of the tendon. UMAP embedding with gene markers and distribution of seven delineated EC populations from human tendons; CD36 high EC (CD36EC), E-Selectin EC (CD62eEC), collagen 4 vessel EC (COL4aEC), immune-like EC (ImmuneEC), LYVE1 positive EC (LYVE1EC) and SEMA3G positive EC (SEMA3GEC). (E) Tenocyte–immune interactions in tendon (n=3 healthy, vs 4 diseased). Predicted cell–cell interactions using CellphoneDB statistical framework on human tendon immune and stromal cells. Selected ligand receptor interactions showing *APP* and *MIF* ligand–receptor pairs in tendon stromal and immune cells. Mean of combined gene expression of interaction pairs (Log2 mean) and p value of specificity of interactions. Violin plots of *APP* and *MIF* expression in tendon stromal cells from healthy (pink) and diseased (green) tendon. Spatial expression (log2FC) of stromal *APP* and macrophage *LYVE1* in normal human tendon and stromal *MIF* and macrophage *CD74* in tendinopathic tendon visualised on 10x Genomics visium data, boxes highlight areas of coexpression. Violin plots of *LYVE1* and *CD74* expression in immune cells from healthy (pink) and diseased (green) tendon. Biorender infographic summarising tenocyte–immune cell interactions in tendon disease. (F) EC–tenocyte interactions in tendon. Predicted cell–cell interactions using CellphoneDB statistical framework on selected human tendon endothelial and stromal cells. Ligand–receptor interactions showing *NOTCH3* ligand–receptor pairs in tendon endothelial and stromal cells. Mean of combined gene expression of interaction pairs (Log2 mean) and p value of specificity of interactions. Violin plots of *NOTCH3* and *JAG1* expression in tendon stromal and ECs, respectively from healthy (pink) and diseased (green) tendon. Spatial expression (log2FC) of *NOTCH3* and *MCAM* from mural tenocytes and *SEMA3G* and *JAG1* from SEMA3GEC’s in human diseased tendon visualised on 10x Genomics visium data, boxes highlight areas of coexpression. Biorender graphic of predicted SEMA3GEC and mT interaction in human tendon.

In healthy (4 biopsies, n=3040 cells) and diseased (5 biopsies, n=19 084 cells) tendon we find a mix of endothelial, immune and stromal cells (figure 1A, online supplemental file 1). Each cell type group is present in disease and healthy tissue but with distinct quantitative and qualitative characteristics. Within stromal populations we identified ‘mural type’ stromal cells (figure 1B). Mural cells, which include pericytes, are possible progenitor cells in tendon^5^ and interestingly, these cells are phenotypically similar to *NOTCH3* high mural cells described in rheumatoid arthritis (RA) synovium which can differentiate into fibroblasts following interactions with endothelial cells (ECs) via *JAG1*.^6^ Cell–cell interaction and S_T_ analysis indicate a similar phenomenon could occur within tendinopathy between mural cells and *SEMA3G* ECs (figure 1F). In all diseased stromal cell populations, there was greater expression of genes for extracellular matrix proteins (eg, *COL1A1*, *COL3A1*, *FN1*, *BGN*) which is considered the hallmark feature of tendinopathy (online supplemental figure S2B). Furthermore, pathway analysis indicates stromal cell clusters shift from negative regulation of immune cell and cytokine responses in normal tendon (online supplemental figure S3A) to a state that promotes immune cell recruitment and activation along with cytokine secretion and response processes in diseased tendon (online supplemental figure S3B, C).

10.1136/annrheumdis-2021-220256.supp1 Supplementary data

10.1136/annrheumdis-2021-220256.supp2 Supplementary data

10.1136/annrheumdis-2021-220256.supp3 Supplementary data

Seven subtypes of *PECAM1*+ ECs were found (figure 1D), including a population of *LYVE1*+ ECs that produce *CCL21* and have been shown to regulate dendritic cell (DC) migration.^7^ Furthermore, *CCL21* is upregulated in these cells in tendinopathy (online supplemental figure S2C). DCs comprise the single largest immune cell population present in normal tendon (figure 1B). Intriguingly, DCs are also present in diseased tendon however, showing therein greater levels of DC activation and lower levels of *C1Q* genes (regulatory DC markers) (online supplemental figure S2D, E). The activation of DCs and subsequent T cell activation^8^ in tendinopathy is further evidenced by pathway analysis of differentially upregulated genes in disease (online supplemental figure S3D, E). This activation may in part be due to increased matrix protein expression, such as *FN1*, which can activate DCs and resulting in alterations in T cell populations within tissue potentially contributing to mechanisms driving disease chronicity. Additionally, we found three populations of macrophages in diseased tendon, one of which, cycling macrophages (figure 1C, online supplemental figure S2A) is unique to diseased tissue. The transcript profile of macrophages in normal tendon most closely resembled tissue repair and debris clearance (figure 1E, online supplemental figure S2D). Within normal tendon we found *APP* expression in tenocytes which can induce a resolution promoting phenotype in macrophages. However, *APP* expression was reduced in the stromal compartment in disease coinciding with diminished expression of *LYVE1* within diseased tissue macrophages (figure 1E, online supplemental figure S2D). These macrophage subsets have recently been associated with RA remission and we postulate the phenotypic drift away from this phenotype promotes aberrant tissue repair and attendant tendinopathy.^9^

Further evidence suggesting that the stromal environment may induce inflammatory changes comprises increased expression of *MIF* (figure 1E, online supplemental figure S2B) in diseased tenocytes that can induce proinflammatory effects via its receptor *CD74*, which is also upregulated in macrophages from diseased tendon (figure 1E, online supplemental figure S2C). S_T_ generated indicative data from cell–cell interaction analysis suggests stromal induced immune regulation. As such, we postulate the primary role of the immune compartment within the tendon is to regulate and resolve damage; however, following cumulative microtrauma the fundamental process of debris removal and matrix repair initiated by tenocytes could lead to positive amplification of the immune compartment. We further propose that within diseased tendon immune homoeostasis may become imbalanced and activated immune cells, primed by both endothelial and stromal cells, promote a cycle of inflammation and aberrant tissue repair. The inflammatory environment, including cytokine pathways that are unequivocally demonstrated in this preliminary tendon atlas, have been targeted to yield potent immunological interventions in a range of inflammatory RMDs—the potential to target and investigate these pathways in human tendon disease is now compelling.
