# Supplementary material for: Single cell and spatial transcriptomics in human tendon disease indicate dysregulated immune homeostasis
Source: Ann Rheum Dis. 2021 May 17;80(11):1494–7. doi: 10.1136/annrheumdis-2021-220256 (PMC8522454; doi:10.1136/annrheumdis-2021-220256)
Supplement: Supplementary data [file annrheumdis-2021-220256supp001.pdf]

Methods

Tissue collection and preparation

All procedures and protocols were approved by the NHS West of Scotland Ethics Committee (REC14/WS/1035) and informed consent was obtained from all patients according to standard procedures. Supraspinatus and tendon samples were collected from patients with rotator cuff tears undergoing shoulder surgery (Table S1). Standardised patient demographics were obtained preoperatively and included age, sex, duration of shoulder symptoms experienced by the patient and the number of subacromial steroid injections. Patients were only included if there was no clinically detectable evidence of subscapularis tendinopathy on a preoperative MRI scan as determined by a musculoskeletal radiologist or macroscopic damage to the subscapularis tendon at the time of arthroscopy as determined by the senior author (NLM)—by these criteria they represented a preclinical cohort. In this cohort, all patients fulfilled the following criteria: (1) a history of shoulder pain and dysfunction, (2) no previous surgery on the affected shoulder, (3) no radiographic sign of fracture of the shoulder and (4) no history of RA or osteoarthritis. Healthy (hamstring) tendon was obtained at the time of routine anterior cruciate ligament (ACL) reconstruction were employed as an independent control group. We acknowledge the limitation of utilising tendon from different anatomical sites. However, obtaining non-diseased shoulder tendon tissue from human subjects is extremely difficult and hamstring tendon has commonly been used in our own and other *in vitro* tendon studies as a surrogate control when comparing to diseased shoulder tendon.

Single-cell RNA-seq

Single-cell suspensions of cells were derived from freshly digested tendon biopsies following surgical excision. Tendon tissue was digested in 0.15mg/ml Liberase TM (Sigma-Aldrich) in 10ml RPMI, kept in constant rotation at 37°C for a maximum of 2 hours. Digested tissue was then filtered and live cells were sorted using a FACS ARIA III. Isolated cells (13561 cells from healthy and 38040 cells from suprapinatus tendon tissue) were lysed and then RNA was reverse-transcribed and converted to cDNA libraries for RNA-seq analysis using a Chromium Controller and Chromium Single Cell 3′ v2 Reagent kit (10x Genomics) following the manufacturer’s protocol. Pooled libraries were used for sequencing on a HiSeq 4000 (Illumina) to a depth of ~30,000

reads per cell. Alignment of reads to the genome and generation of gene counts per cell were performed by Cell Ranger software (10x Genomics). 4080 cells from healthy and 22004 cells from supraspinatus tendon tissue were sequenced. Quality control was performed on each sample and poor-quality cells were removed on the basis of number of genes expressed (<200), of unique molecular identifiers (UMIs) and percentage of mitochondrial reads mapped (>5%). Following this QC, we normalised and scaled the data using Seurat v4.0 package (Sajita Lab) for all the cells (health k= 3040, supraspinatus k=19084). Then principal components analysis and high-quality cells were clustered using a graph-based routine implemented in Seurat R package and its integration method for multiple samples (Satija Lab). All cells from the tissue were clustered and individual cell types were computationally isolated for further analysis, including cluster markers and differential gene expression.

Cell–cell interactions

Using the cluster markers found from Seurat we ran CellPhoneDB as follows: cellphonedb method statistical_analysis meta.tsv counts.tsv– counts-data = gene_name–threads = 60. CellPhoneDB raw predictions were filtered by removing those interactions with a P > 1.0 × 10−5. Significant pairs were then filtered for the most significant predicted interactions.

Gene Ontology analysis

Gene ontology (GO) analysis was conducted by generating cluster markers and differentially expressed genes using Seurat as described above. The list of genes was then input into STRING (https://string-db.org) for functional enrichment anlysis.

Spatial transcriptomics

Visualisation of gene expression within tendon tissue was conducted using 10x Visium spatial gene expression kit(10x Genomics) as per manufacturers protocol on independent tendon tissue from samples used for single-cell RNA-seq. Briefly, additional healthy (n=3) and diseased tendon (n=4), collected as above, were immediately embedded in Optimal Cutting Compound (OCT) media and frozen in liquid-nitrogen-cooled isopentane bath, cut into 10µm sections using Thermo Scientific CryoStar cryostat, and mounted on 10X Visium slides, which were pre-cooled to −20°C. Slide were stained for H&E and then sections were imaged using Zeiss PALM

MicroBeam laser capture microdissection system and the images were stitched together using Zeiss software. The sections were then permeabilised for 10 minutes and spatially tagged cDNA library cDNA libraries constructed using the 10x Genomics Visium Spatial Gene Expression 3' Library Construction V1 Kit. cDNA libraries were sequenced on an Illumina NextSeq 500/550 using 150 cycle high output kits with sequencing depth of ~5000 reads per spot. Sequencing data and images were aligned using the Space Ranger 1.0.0 pipeline to derive a feature spot-barcode expression matrix (10X Genomics). Seurat 4.0 spatial expression workflow (https://satijalab.org/seurat/articles/spatial_vignette.html) was adopted to integrate, log-transform and normalised data before plotting genes of interest on section.
