# Supplementary figures and images for: Single cell and spatial transcriptomics in human tendon disease indicate dysregulated immune homeostasis
Source: Ann Rheum Dis. 2021 May 17;80(11):1494–7. doi: 10.1136/annrheumdis-2021-220256 (PMC8522454; doi:10.1136/annrheumdis-2021-220256)

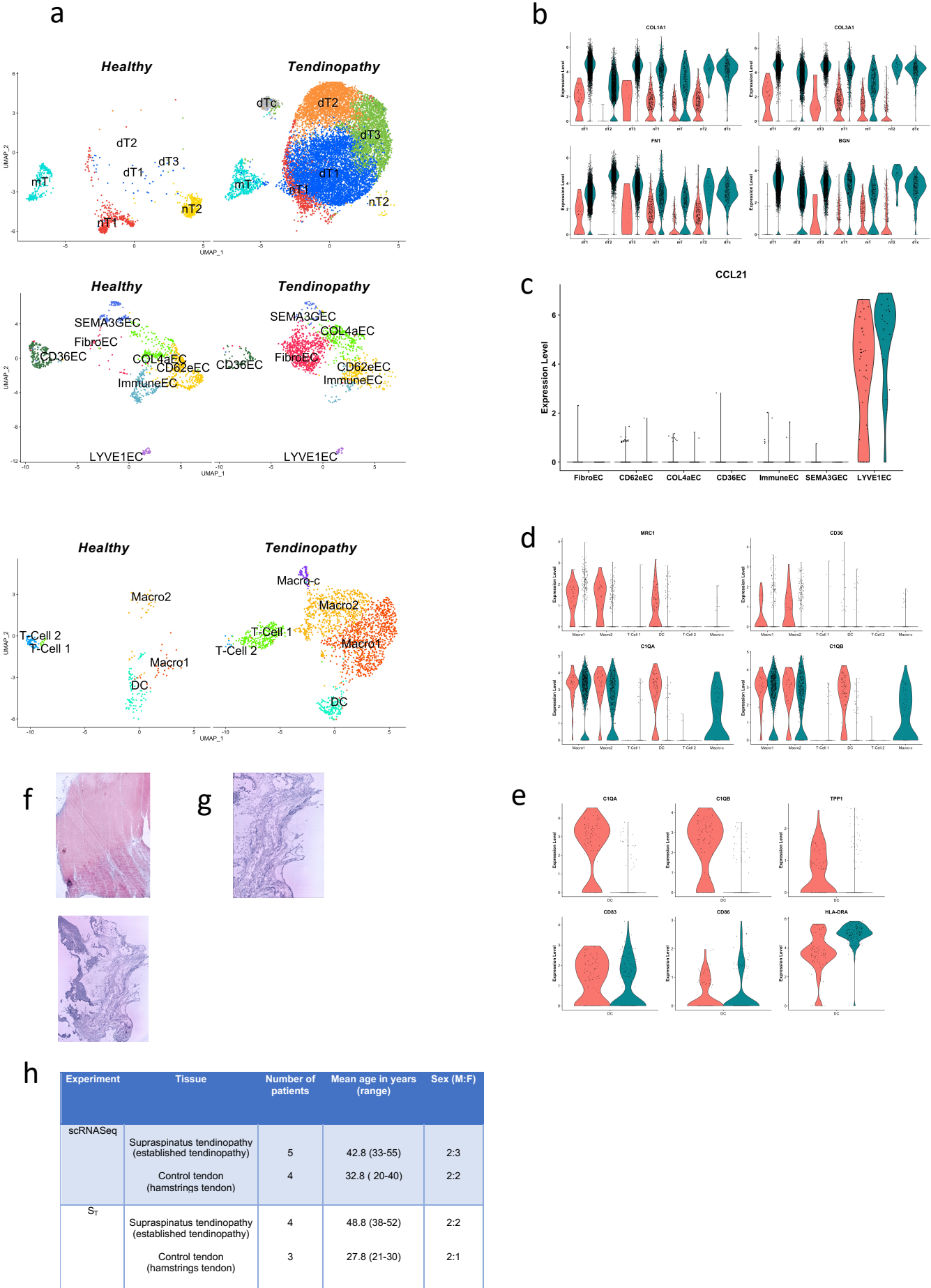

Supplement: Supplementary data [file annrheumdis-2021-220256supp002.pdf]

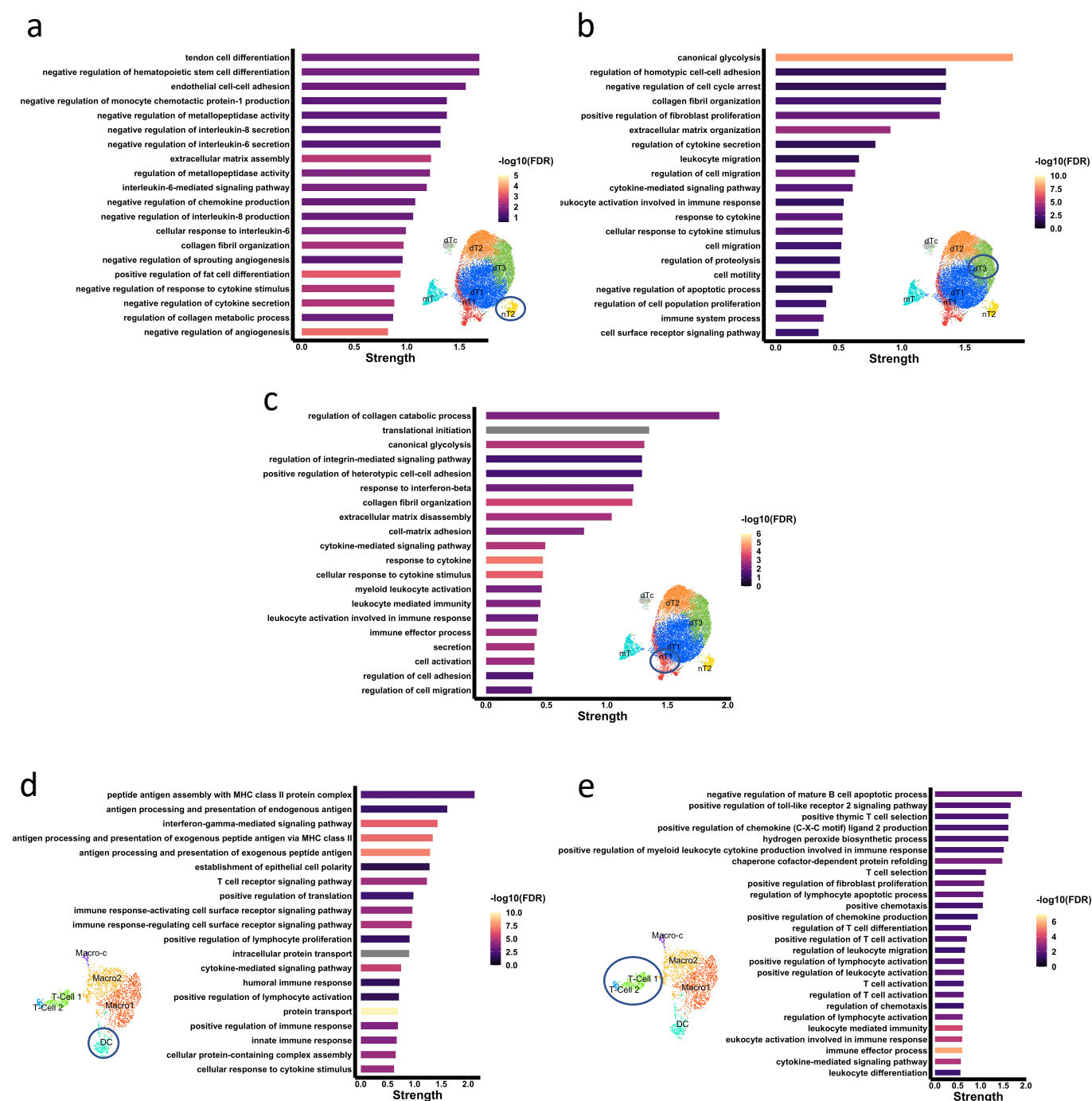

Supplement: Supplementary data [file annrheumdis-2021-220256supp003.pdf]
